# Ultrasound-guided trans-rectal high-intensity focused ultrasound (HIFU) for advanced cervical cancer ablation is feasible: a case report

**DOI:** 10.1186/s40349-015-0043-6

**Published:** 2015-12-18

**Authors:** M. Abel, H. Ahmed, E. Leen, E. Park, M. Chen, H. Wasan, P. Price, L. Monzon, W. Gedroyc, P. Abel

**Affiliations:** Department of Surgery & Cancer, Imperial College London, Hammersmith Hospital, Du Cane Road, London, W12 0HS UK; Department of Urology, University College Hospital, 235 Euston Road, London, NW1 2BU UK; Department of Radiology, Imperial College London, Hammersmith Hospital, Du Cane Road, London, W12 0HS UK; Department of Oncology, Imperial College Healthcare NHS Trust, Hammersmith Hospital, Du Cane Road, London, W12 0HS UK; Department of Radiology, Guy’s and St Thomas’ Hospital NHS Trust, St Thomas’ Hospital, Westminster Bridge Road, London, SE1 7EH UK; Department of Radiology, Imperial College London, St Mary’s Hospital, Praed Street, London, W2 1NY UK

**Keywords:** Cancer, Cervical, Gynaecology, Intensity, Focused, Ultrasound, FUS, HIFU

## Abstract

**Introduction:**

High-intensity focused ultrasound (HIFU) is an ablative treatment undergoing assessment for the treatment of benign and malignant disease. We describe the first reported intracavitary HIFU ablation for recurrent, unresectable and symptomatic cervical cancer.

**Case description:**

A 38 year old woman receiving palliative chemotherapy for metastatic cervical adenocarcinoma was offered ablative treatment from an intracavitary trans-rectal HIFU device (Sonablate® 500). Pre-treatment symptoms included vaginal bleeding and discharge that were sufficient to impede her quality of life. No peri-procedural adverse events occurred. Symptoms resolved completely immediately post-procedure, reappeared at 7 days, increasing to pre-procedural levels by day 30.

**Discussion and evaluation:**

This first time experience of intracavitary cervical HIFU suggests that it is feasible for palliation of advanced cervical cancer, with no early evidence of unexpected toxicity. Ethical approval had also been granted for the use of per-vaginal access if appropriate. This route, alone or in combination with the rectal route, may provide increased accessibility in future patients with a redesigned device more suited to trans-vaginal ablations.

**Conclusion:**

Intracavitary HIFU is a potentially safe procedure for the treatment of cervical cancer and able to provide symptomatic improvement in the palliative setting.

## Background

High-intensity focused ultrasound (HIFU) is a non-invasive ablative treatment undergoing assessment for the treatment of benign and malignant disease with encouraging results. As technology advances and expertise accumulates, previous limiting factors including image-guidance (to accurately identify, target and monitor treatment) and lengthy therapeutic duration are being overcome. We describe the first reported intracavitary HIFU ablation for cervical cancer and determine that HIFU was feasible with demonstrable benefits.

Cervical cancer is the fourth most common cancer in women worldwide, with an estimated incidence of approximately 528,000 diagnoses and 266,000 deaths annually [1]. It is potentially curable with a multi-modal approach that may include surgical excision, radiotherapy and cytotoxic chemotherapy. These therapeutic modalities may also delay progression and improve symptom control in advanced disease by palliating the unpleasant symptoms that include vaginal bleeding and/or discharge and intractable pain which can all impact negatively on quality of life.

HIFU conveys sonic energy to a defined target raising temperature to cause cell death by coagulative necrosis. Single-element devices consist of a curved transducer or incorporate lens to focus the energy at the target site. Ultrasonic waves from multi-element arrays converge at the treatment zone to cause ablation. Intervening tissues remain completely unharmed. Ablations are sequentially repeated in adjacent pathological tissue until the desired volume is destroyed. Guidance and monitoring of the treatment is through integrated ultrasound or magnetic resonance imaging [2].

HIFU devices are either intracavitary (minimally invasive through insertion into rectum or vagina) or extracorporeal (completely non-invasive). Intracavitary devices in the rectum have been extensively studied in thousands of men with prostate cancer [3], but to date, there has been no report of intracavitary HIFU in cervical cancer. Extracorporeal HIFU has been reported in one woman with cervical cancer successfully palliating local pain [4]. In the past, lengthy treatment times and difficulties with accuracy of targeting have prevented wide-spread HIFU use, although current technology is rapidly improving to overcome these limitations.

UK National Ethics Committee approval (09/H0808/43) was granted for a pilot study to assess the feasibility, safety and efficacy of ultrasound-guided intracavitary HIFU in pelvic malignancy. Intracavitary HIFU treatment of a man with recurrent anastomotic colorectal cancer by our group resulted in symptomatic improvement within 24 h and radiological evidence of tumour necrosis at the target ablation site [5]. We wanted to expand the potential of intracavitary HIFU therapy by, for the first time, determining feasibility for its use and ability to palliate, relieve pain and improve quality of life in advanced cervical cancer.

## Case presentation

A 38 year old woman presenting with stage 1B2 histologically confirmed cervical adenocarcinoma and received primary chemotherapy/radiation followed by a brachytherapy boost. Ten months later, a recurrent local cervical mass and small volume nodal and pulmonary disease were identified radiologically. Palliative carboplatin and paclitaxel was given, but follow-up imaging identified continued disease progression and she remained with a persistent continuous, offensive smelling vaginal discharge requiring 7–8 pads daily and impacting her quality of life.

Chemotherapy was stopped 4 months prior to the ablation. Pre-HIFU treatment magnetic resonance imaging measured the cervical mass at approximately 7 × 4 × 4 cm, with invasion into adjacent fat planes and the lower myometrium. A computed tomography scan identified intralesional gas; although, a fistulous connection was ruled out on physical examination.

### HIFU procedure

Fully informed, written consent was obtained prior to enrollment in this study. Under general anaesthetic, an intracavitary-guided HIFU device (Sonablate® 500, Sonacare Medical, Charlotte, NC, USA) currently utilised in the treatment of prostatic malignancy was passed into the rectum, as adequate alignment with the tumour could not be obtained trans-vaginally. The tumour and surrounding structures were visualised using integrated ultrasound imaging in sagittal and cross-axial planes, also providing treatment monitoring. Intralesional gas obscured the majority of the cranial portion of the lesion. Ultrasound energy cannot penetrate air so HIFU was targeted to an approximately 4 × 4 × 1 cm region at the inferior margin, the most likely area causing symptoms.

**Fig. 1:**
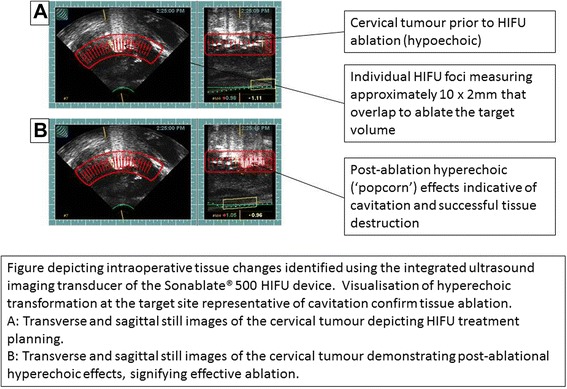
Intraoperative tissue changes. Figure depicting intraoperative tissue changes identified using the integrated ultrasound imaging transducer of the Sonablate® 500 HIFU device. Visualisation of hyperechoic transformation at the target site confirms tissue ablation. **a** Transverse and sagittal still images of the cervical tumour depicting HIFU treatment planning. **b** Transverse and sagittal still images of the cervical tumour demonstrating post-ablational hyperechoic effects, signifying effective ablation

Hyperechoic greyscale changes and gas formation, representing ablative effects [6], were visualised using the real-time integrated ultrasound imaging to monitor for the presence of tissue destruction (Fig. 1). Additional, trans-abdominal ultrasound imaging was performed using an iU22 (Philips Healthcare, Bothell, WA, USA) with a curvilinear C5-1 transducer during treatment pauses for monitoring targeting accuracy and treatment success. This was used to supplement available trans-rectal imaging in order to more closely observe the procedure. To minimise potential toxicity, the lowest energy input required to cause cell death was used during the procedure. This was determined via integrated imaging, by monitoring for hyperechoic changes. Tissue changes were observed with output powers of 33 W/cm^2^ per individual foci, lower than the 40 W/cm^2^ required for prostatic HIFU therapy. Total treatment time was 40 min.

### Post-procedure outcome

**Fig. 2:**
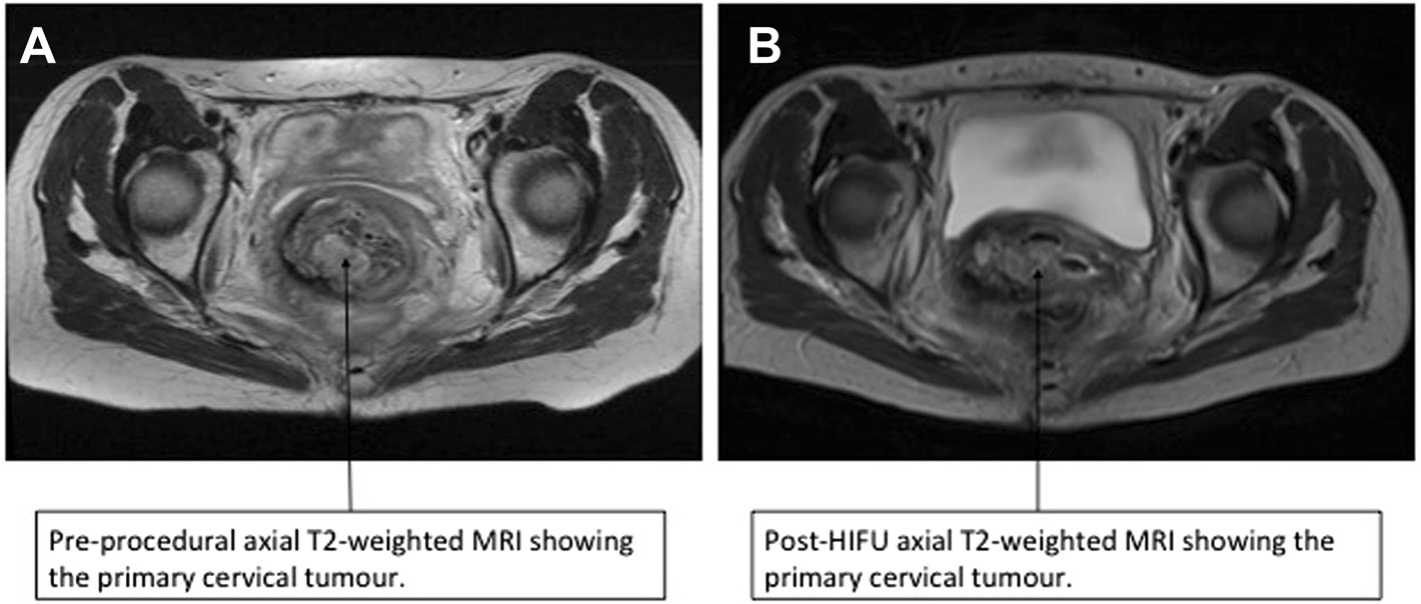
Pre- and post-treatment MRI changes. Axial T2-weighted MRI at the level of the pelvic side wall showing the primary cervical tumour at baseline (**a**) and post-treatment (**b**). A significant tumour burden remains, although, the AP length of the lesion has reduced in size from 4.5 to 3.4 cm

No intra- or immediate post-procedural adverse events occurred. Upon waking from the anaesthetic, vaginal bleeding and discharge had stopped completely. The patient was discharged home within 24 h of the procedure, after a precautionary overnight stay to monitor for unexpected adverse events. She reported mild lower abdominal pain after 48 h, which settled with simple analgesia (1 g oral paracetamol, four times daily). At 7 days, vaginal bleeding and discharge restarted at a lower volume than in pre-treatment and reverted to pre-procedural levels by 30 days. Baseline and post-therapy imaging is shown in Fig. 2. Despite clinical benefit, there was no significant reduction in lesion size following HIFU ablation, although this was not unexpected as only a small portion of the tumour was treated. The patient received palliative therapy for symptomatic relief under the guidance of the oncologists.

### Discussion and evaluation

This first time experience of intracavitary cervical HIFU suggests that it is feasible for palliation of advanced cervical cancer, with no early evidence of unexpected toxicity. Rapid symptomatic benefit was achieved that has improved the quality of life. In contrast to radiotherapy, HIFU offers the additional benefit of no dose ceiling, allowing for repeated re-treatments if deemed appropriate on an individual basis.

Ethical approval had also been granted for the use of per-vaginal access if appropriate. Although considered for the procedure, insertion of the transducer into the vagina did not prove possible in this case. The device was not initially intended for gynaecological use, and it proved impossible to obtain satisfactory alignment to the target site as energy delivery is perpendicular to the transducer. This demonstrates the need to have HIFU transducers specifically tailored to individual indications. Alone or in combination with the rectal route, this may provide increased accessibility in future patients with a redesigned device more suited to trans-vaginal ablations. Trans-vaginal ablations would also reduce the risk of post-procedural fistulae when compared to the trans-rectal approach, remove the possibility of skin burns seen with trans-abdominal treatments and lower the potential for damage to the healthy intervening tissue, by shortening the distance to the tumour.

## Conclusions

In conclusion, further detailed studies are now required to fully assess safety and efficacy of HIFU, as well as dosimetry and development of a dedicated cervical/gynaecological probe design. As HIFU technology progresses, it could provide minimal toxicity with good relief of symptoms in patients who otherwise may have limited treatment options. In the future, HIFU could also become an established part of a multi-modal approach with curative intent in combination with current available therapy options to treat early stages of cervical and other gynaecological cancers.
